# Supplementary material for: In vitro fermentation characteristics of polysaccharide from Scrophularia ningpoensis and its effects on type 2 diabetes mellitus gut microbiota
Source: PeerJ. 2025 May 5;13:e19374. doi: 10.7717/peerj.19374 (PMC12060902; doi:10.7717/peerj.19374)
Supplement: Supplemental Information 1 [file peerj-13-19374-s001.docx]

**Table S1 Preparation of simulated digestion solution.**

|  | **Simulated gastric buffer (mmol/L)** | **Simulated small intestinal buffer (mmol/L)** |
| --- | --- | --- |
| KCl | 6.9 | 6.8 |
| KH_2_PO_4_ | 0.9 | 0.8 |
| NaHCO_3_ | 25 | 85 |
| NaCl | 47.2 | 38.4 |
| MgCl_2_·6H_2_O | 0.1 | 0.33 |
| (NH_4_)_2_CO_3_ | 0.5 | - |
| pH | 3 | 7 |
