# Supplementary figures and images for: In vitro fermentation characteristics of polysaccharide from Scrophularia ningpoensis and its effects on type 2 diabetes mellitus gut microbiota
Source: PeerJ. 2025 May 5;13:e19374. doi: 10.7717/peerj.19374 (PMC12060902; doi:10.7717/peerj.19374)

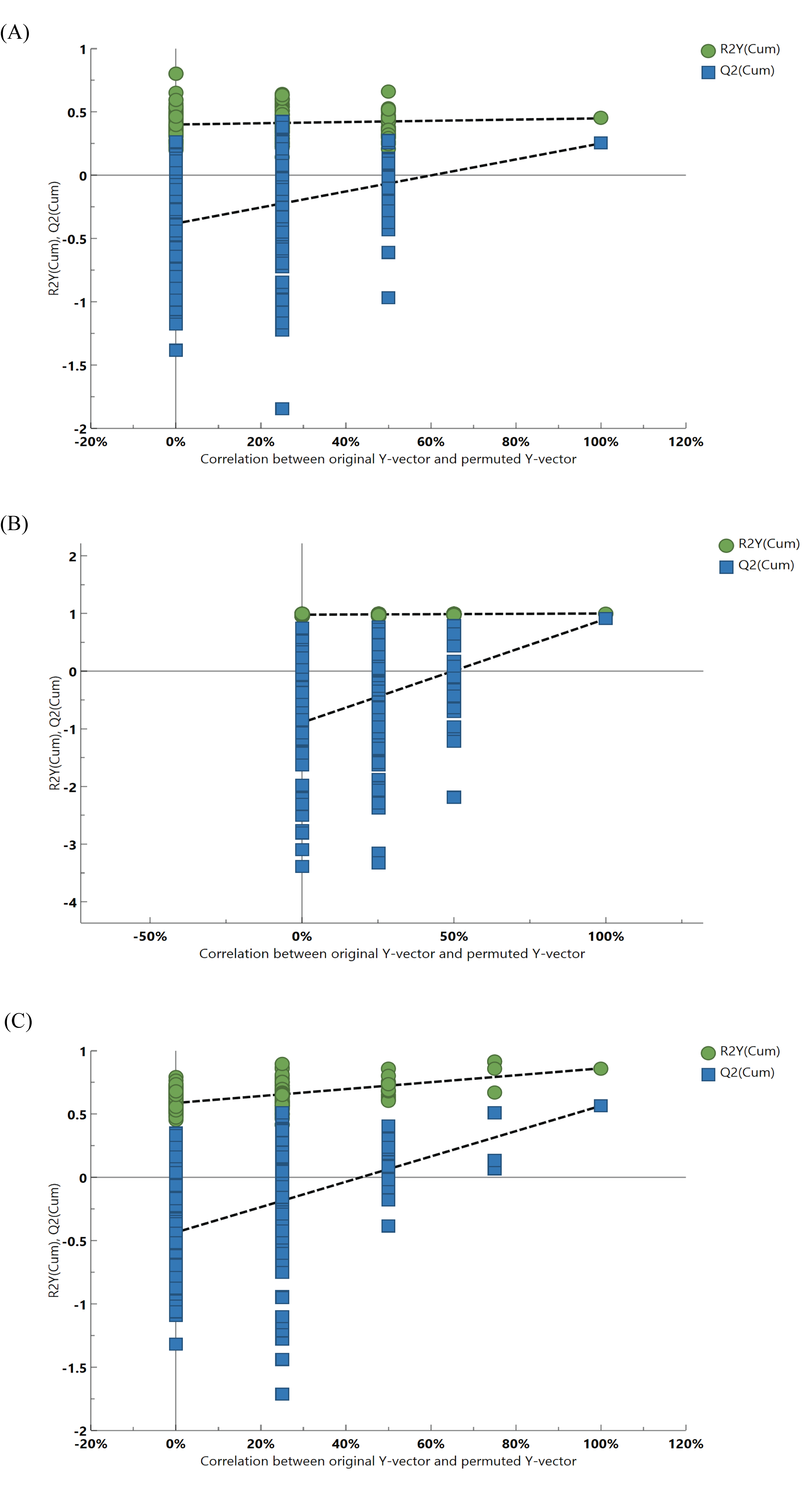

Supplement: Supplemental Information 4 — (A) T2DM group vs. HC group; (B) HC+SNP group vs. HC group; (C) T2DM+SNP group vs. T2DM group. [file peerj-13-19374-s004.png]
